# Terahertz Spectroscopy Unambiguously Determines the Orientation of Guest Water Molecules in a Structurally Elusive Metal–Organic Framework

**DOI:** 10.1021/acs.jpclett.4c00706

**Published:** 2024-05-16

**Authors:** Saheed A. Ajibade, Luca Catalano, Johanna Kölbel, Daniel M. Mittleman, Michael T. Ruggiero

**Affiliations:** † Department of Chemistry, University of Vermont, Burlington, Vermont 05405, United States; ‡ Department of Chemistry, University of Rochester, Rochester, New York 14627, United States; ¶ Department of Life Sciences, University of Modena and Reggio Emilia, 41125 Modena, Italy; § School of Engineering, Brown University, Providence, Rhode Island 02912, United States

## Abstract

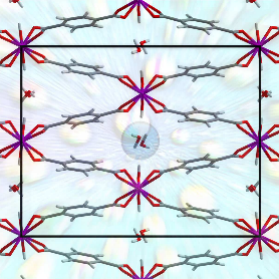

Porous materials, particularly metal–organic frameworks (MOFs), hold great promise for advanced applications. MIL-53(Al) is an exceptionally well-studied MOF that exhibits a phase transition upon guest capture—in this case, water—resulting in a dramatic change in the pore volume. Despite extensive studies, the structure of the water-loaded narrow-pore phase, MIL-53(Al)-*np*, remains controversial, particularly with respect to the positions of the adsorbed water molecules. We use terahertz spectroscopy, coupled with powder X-ray diffraction and density functional theory simulations, to unambiguously resolve this controversy. We show that the low-frequency (<100 cm^–1^) vibrational spectrum depends on weak long-range forces that are extremely sensitive to the orientation of the adsorbed water molecules. This enables definitively determining the correct structure of MIL-53(Al)-*np* while highlighting the extreme sensitivity of terahertz spectroscopy to bulk structure, suggesting its potential as a robust complement to X-ray diffraction for precise characterization of host–guest complexes.

Porous materials represent one of the most exciting areas of materials science, as they hold potential to revolutionize a number of different areas of chemistry, ranging from gas storage to drug delivery.^1−5^ One important area that has shown great promise involves passive water harvesting for clean water generation.^6−8^ Researchers have described a number of different materials in recent years that are designed to uptake atmospheric water at one relative humidity while releasing it at a different (often lower) humidity.^6,9,10^ For metal–organic frameworks (MOFs) in particular, the uptake parameters are optimized to work in a particular environment such that these materials offer a robust, economical, and energy-efficient approach to producing clean water.^11,12^ In order to better understand and design such systems, one requires a detailed understanding of the structure and dynamics of the frameworks, with and without water present, as it is well-known that the location and orientation of guest molecules can have a profound impact on bulk physical properties. However, identifying the structure of guest molecules turns out to be a significant challenge.^13,14^ It requires the growth of large single-crystal samples, and in the case of water specifically, it likely requires neutron diffraction measurements in order to locate the hydrogen atoms, as X-rays are poorly diffracted by protons.^15−17^ For MOFs in particular, this is especially difficult, as many MOFs only form submicrometer-sized crystallites, which typically can only be characterized with powder diffraction measurements. And while PXRD can be utilized for full structural determination, often via the Rietveld method, the positions of hydrogen atoms cannot in general be reliably extracted from these measurements. Thus, methods that can provide insight into the structure of guest molecules without these constraints are sorely needed.

MIL-53(Al) is an extremely well-studied MOF system that is known to exhibit a range of interesting structural effects. It has been reported to exist in four distinct phases that are strongly influenced by a range of stimuli such as temperature, pressure, and the presence (or absence) of guest molecules.^18−20^ They are the generic as-synthesized phase; the high-temperature large-pore phase, obtained upon calcination of the as-synthesized form; the low-temperature narrow-pore phase, which results from cooling and adsorption of water molecules at room conditions;^21^ and the closed-pore phase (with no guest molecule), which is achieved either by high-pressure^22,23^ or low-temperature treatment of the large-pore form.^24^ The structural flexibility of MIL-53(Al) has primed it for many varied advanced applications as a smart and adaptive porous material.^19^ At room temperature, it undergoes a reversible transition from the large-pore to the narrow-pore phase through a breathing effect by adsorption of water molecules into the framework,^18,25,26^ causing a significant volume fluctuation of up to 40%. This absorption-influenced transition is not limited to water alone, as other guest molecules, including CH_4_, N_2_, CO, and O_2_, have been shown to initiate a similar effect.^27−29^ Additionally, the stability of MIL-53(Al) under high mechanical stress has also been noted,^30^ as it has been reported to undergo a pressure-induced phase transition from its large-pore form to the narrow-pore form at high pressures suggesting its suitability as a nanoshock absorber.^23^ Further, MIL-53(Al) is environmentally and biologically friendly.^31−33^ Overall, the interesting guest-induced framework dynamics in MIL-53(Al) are important to understand from a fundamental standpoint, in order to further design and tune the adsorption behavior of host–guest complexes, and this represents a model system for studying the broader class of flexible MOFs.

Despite this broad interest, there remain many inconsistencies in the literature regarding the structure of the narrow-pore phase of MIL-53(Al) (MIL-53(Al)-*np*). Loiseau et al. first synthesized and characterized it using powder X-ray diffraction in 2004 and reported it as a monoclinic crystal belonging to the *Cc* space group, with lattice parameters of *a* = 19.513 Å, *b* = 7.612 Å, *c* = 6.576 Å, and β = 104.24°, containing only a single symmetry-independent water molecule in the pore (Figure 1).^21^ These conclusions were supported by Seoane et al., who also used X-ray powder diffraction.^34^ A different study used density functional theory (DFT) optimizations to suggest that MIL-53(Al)-*np* crystallized in the *C*2/*c* space group but with cell parameters similar to those previously reported by Loiseau et al.^35^ However, a combination of nuclear magnetic resonance (NMR) spectroscopy and PXRD suggested that there were actually two symmetry-independent pairs of water molecules in two distinct pores, resulting in a *P*2_1_/*c* space group with a doubled unit cell compared to the previous structures, with cell parameters of *a* = 19.5042 Å, *b* = 15.2014 Å, *c* = 6.5693 Å, and β = 104.18° (Figure 1).^36^ This same result was later confirmed by Wong-Ng et al., also using powder X-ray diffraction.^37^ These varied results highlight the difficulties in definitively determining the accurate and complete structure of MIL-53(Al)-*np* using PXRD methods and showcase the need for complementary techniques that can effectively and unambiguously determine the structure of these types of systems. This is especially true for researchers who require accurate structures in order to perform further downstream analyses, such as DFT simulations for properties such as elasticity. Incorrectly identifying the location of guest molecules (and associated intermolecular contacts such as hydrogen bonding) will surely lead to inaccurate computed results. In fact, in some cases, the ambiguity of the water positioning in MIL-53(Al)-*np* has precluded it from being studied computationally.^38^

**Figure 1:**
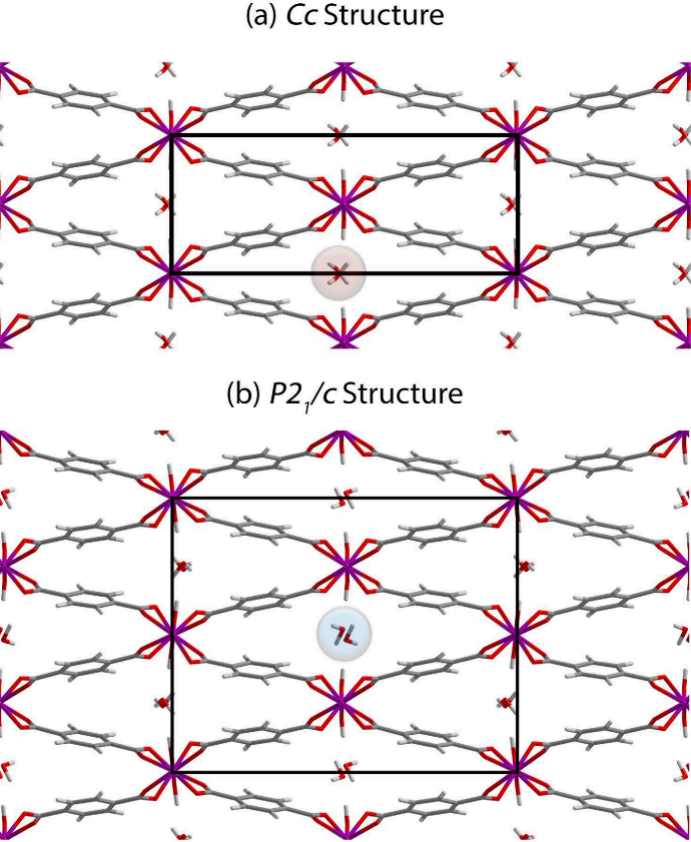
Structures of MIL-53(Al)-*np*. (a) The well-referenced *Cc* structure with only one pore per unit cell, and (b) the DFT-optimized *P*2_1_/*c* structure suggested here, with two pores per unit cell.

In order to unambiguously determine the structure of MIL-53(Al)-*np* in the narrow-pore phase, we use a combination of PXRD, THz spectroscopy, and DFT simulations. In particular, because of its extreme sensitivity to minor changes in condensed phase structures, THz vibrational spectroscopy is an ideal tool for determining the configuration of water molecules in the pore of MIL-53(Al)-*np*. Indeed, low-frequency vibrational spectroscopy covering the terahertz range (0.1–5 THz, 3–166 cm^–1^) is a powerful and very general technique for probing long-range weak forces in condensed phase materials.^39−42^ The phonon modes at these energies often involve motions of entire molecules or large portions of molecules, which explore significant regions of the intermolecular potential energy hypersurface.^43^

The long-range nature of the forces involved in terahertz dynamics makes the technique a powerful tool for studying structural changes in crystalline solids.^44^ This is because any alteration of the bulk packing of molecules in a crystal will reconfigure the set of weak forces that act on individual molecules, which in turn results in a restructuring of the low-frequency vibrational dynamics.^45,46^ This sensitivity has been demonstrated extensively for the detection of different polymorphic materials, for quantifying crystalline content in amorphous materials, and, recently, as a powerful complement in crystal structure prediction studies.^47^ Porous materials have also been studied with terahertz spectroscopy,^48−55^ including in situ studies of gas adsorption in organic clathrates as a function of both temperature and (gaseous) pressure, which can offer quantitative measurements of loading fraction due to the dramatic changes in the spectra as gas adsorbs into the material.^56−58^

Although some earlier work has investigated the low-frequency vibrational modes of MIL-53(Al), none have explored the regime below 100 cm^–1^. Hoffman et al. investigated the evacuated closed-pore and large-pore structures of MIL-53(Al) with Raman spectroscopy,^59^ and a second work from the same group reported the experimental far-IR spectrum of MIL-53(Al)-*np* from 100–700 cm^–1^.^38^ Additionally, Titov et al. reported the dielectric spectrum of MIL-53(Al)-*np* using synchrotron radiation down to ca. 100 cm^–1^.^60^ Our results demonstrate that the regime below 3 THz (100 cm^–1^) is the most relevant for probing host–guest structure and dynamics.

We first synthesized the material according to established literature methods.^21^ Briefly, aluminum nitrate nonahydrate and terephthalic acid were combined in a 2:1 molar ratio in deionized water and heated in a Teflon-lined stainless steel bomb for 3 days at 220 °C. The resulting solid was washed with water and purified by heating at 330 °C for 3 days in ambient conditions to remove excess terephthalic acid, which produced pure MIL-53(Al)-*np*, confirmed by PXRD. The experimental PXRD pattern is shown in Figure 2.

**Figure 2:**
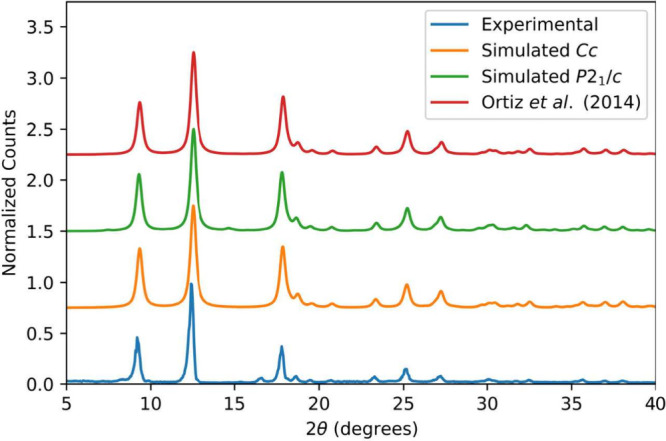
Experimental (blue) and simulated PXRD patterns for MIL-53(Al)-*np*.

In addition to the experimental structure, we fully optimized the structure of MIL-53(Al) using solid-state DFT simulations. The optimizations were initially performed without any constraints other than maintaining the space group symmetry of the solid, meaning that all atomic positions and lattice vectors were allowed to relax. Initially, the well-established structure of MIL-53(Al)-*np* in the *Cc* space group was utilized as the basis of the calculations, and the DFT-optimized and experimental PXRD patterns were in excellent agreement, as shown in Figure 2. Importantly, optimizations performed in other symmetries yielded nearly identical PXRD patterns, implying that it is very difficult to discern which structure is correct based on PXRD analysis alone (*vide infra*).

For terahertz measurements, samples were prepared by gently grinding MIL-53(Al)-*np* using a mortar and pestle and were also characterized using powder X-ray diffraction to ensure bulk homogeneity and sample purity. The resulting powders were mixed with polytetrafluorethylene (PTFE) to a 5% w/w concentration, followed by pressing into free-standing pellets using a hydraulic press. To ensure the applied pressure did not alter the sample composition, we confirmed the structure of MIL-53(Al) with PXRD prior to and after pressing into pellets.

The cryogenic (50 K) terahertz spectrum of MIL-53(Al)-*np* is shown in Figure 3 in both panels in blue. It is clearly obvious that the simulated spectrum of the structure constrained to the *Cc* space group does not match the experimental one, not only in the location of the absorption features but also in the total terahertz vibrational density of states. This implies that there are significant inaccuracies in the calculation, most likely implying that the utilized structure is incorrect. It is important to note that this structure has been used previously to assign the infrared spectrum of MIL-53(Al)-*np* above 100 cm^–1^; it is only at these lower frequencies that the deficiencies of the structural model are revealed.^60^

**Figure 3:**
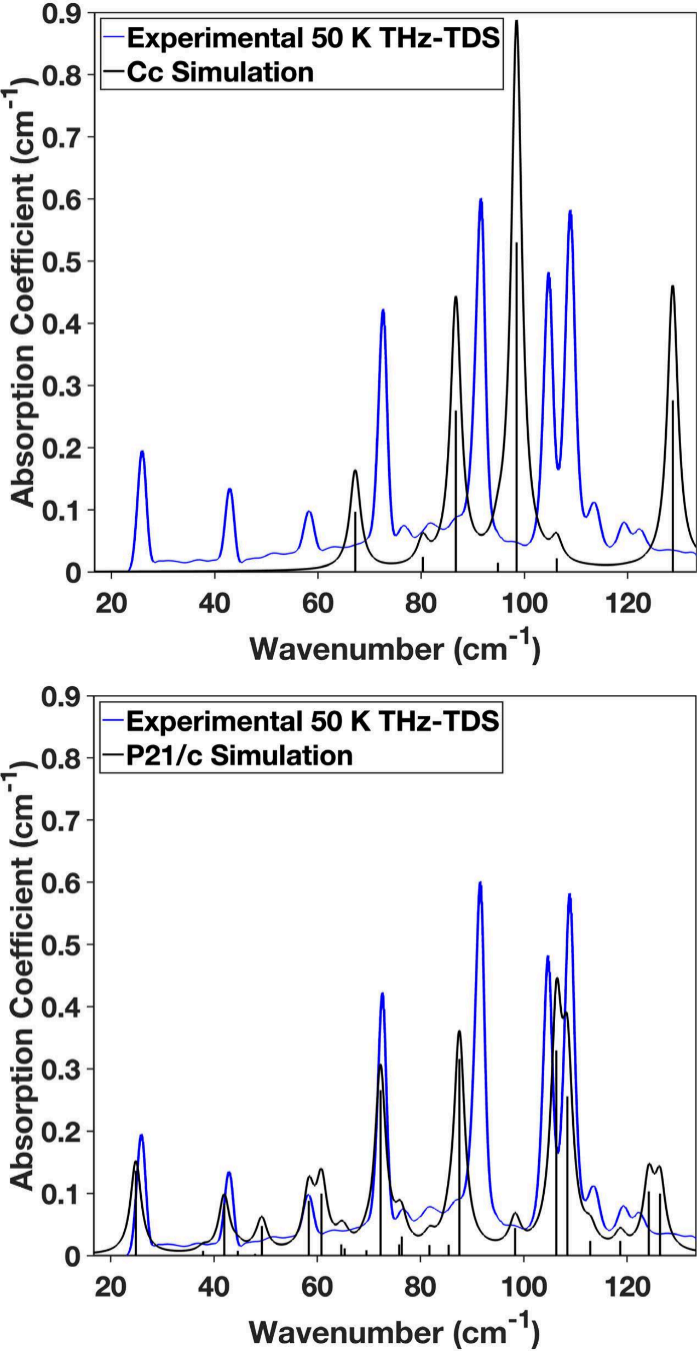
50 K experimental THz-TDS spectrum (blue) compared to the simulated spectra in the *Cc* (top) and *P*2_1_/*c* (bottom) space groups.

In order to investigate other potential structures for MIL-53(Al)-*np*, we proceeded along two fronts. As we originally did not find the proposed *P*2_1_/*c* structure in our initial literature search, we performed a fully *ab initio* structure search to find the lowest-energy structure. This was accomplished by randomly placing two water molecules in the pore of MIL-53(Al)-*np* using the *Cc* structure but in the absence of any space group symmetry, i.e., *P*1, which contained one pore per unit cell. We also generated supercells by sequentially doubling the unit cell along each lattice vector, creating three different templates: 2 × 1 × 1, 1 × 2 × 1, and 1 × 1 × 2. These yielded two pores per unit cell, meaning that four water molecules were randomly placed within the structures, again in the absence of space group symmetry. These models were then fully optimized using solid-state DFT. Many of these optimizations converged to a single common structure, which contained two pairs of water molecules. The lowest-energy structure was subjected to a symmetry analysis, which yielded a structure in the *P*2_1_/*c* space group, which was then subsequently reoptimized within the space group symmetry constraint. The PXRD pattern for this structure is shown in Figure 2 and is also in excellent agreement with the experimental pattern. As noted above, we emphasize that there is very little difference between this new *P*2_1_/*c* and the original *Cc* PXRD pattern. Indeed, the lattice parameters and framework structures are very similar between the two cases, with the only difference being the orientation of the water molecules within the pores, with the important caveat that there are two symmetry-independent water molecules in the *P*2_1_/*c* structure while only one in the *Cc* structure.

Additionally, we then used the structure proposed by Ortiz et al.^36^ as the basis for a geometry optimization. The PXRD pattern for the *P*2_1_/*c* structure reported by Ortiz is shown in Figure 2. Again, the pattern for that structure is in good agreement with all of the other PXRD patterns. However, the orientation of the water molecules in the reported structure differed from what was found using the DFT optimizations. Importantly, optimizing the structure from Ortiz yields the same structure as what was found from the blind structural search, lending some confidence that this newly proposed structure is the correct one. To further confirm this, we performed *ab initio* molecular dynamics (AIMD) simulations to track the water dynamics. The reported Ortiz structure was used as the starting point for the simulation, and the calculations were performed without any symmetry constraints within the NVT ensemble. The simulation ran for 20 000 steps (0.5 fs per step, 10 ps total), and upon completion, the orientations of the water molecules were determined by measuring the angle formed between the plane of the atoms in each water molecule to the horizontal and vertical unit cell planes, for each frame of the trajectory, and histograms for each angle were generated. It was obvious that there were two pairs of symmetry-related (inversion) water molecules, and therefore, only angles for the symmetry-independent water molecules are shown in Figure 4. The vertical lines in Figure 4 represent the static orientation of the water molecules after a complete geometry optimization (solid lines) and those reported by Ortiz (dashed lines). It is clear that while the structure reported by Ortiz has water positioning that is similar to the most frequently observed orientation of the water molecules in the AIMD simulation, the completely optimized structure exhibits a better description of the overall structure, lending further confidence that the proposed structure is indeed the correct one.

**Figure 4:**
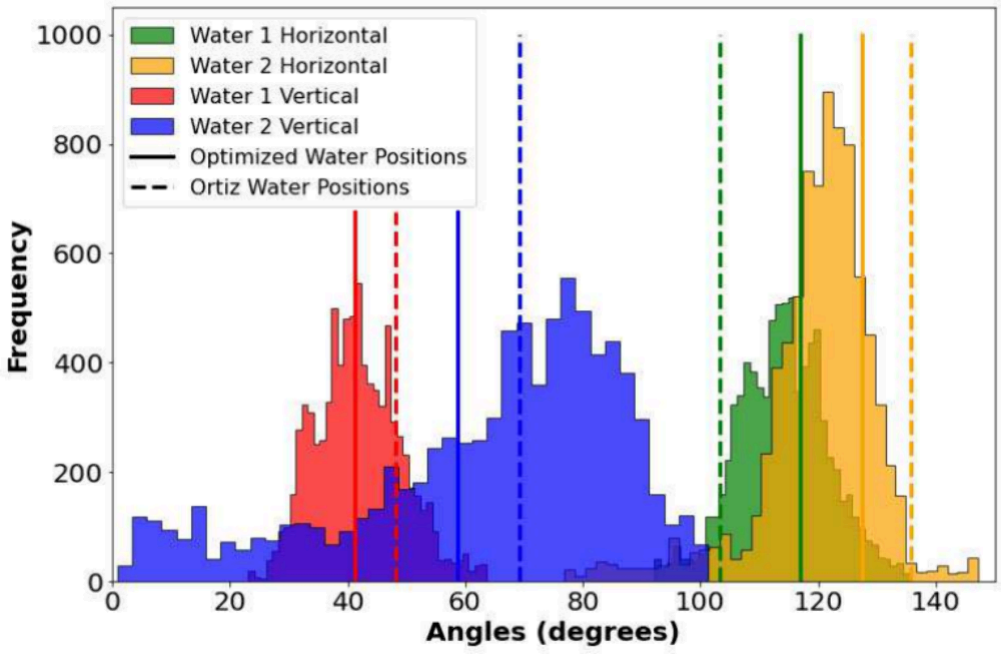
Distribution of the orientation of the symmetry-independent water molecules from AIMD simulations, and the corresponding static orientation of the *P*2_1_/*c* water molecules from Ortiz^36^ (dashed vertical lines) and from the DFT-optimized structure (solid vertical lines). The latter provides a better match to the AIMD distributions.

Bolstered by what appears to be the identification of the correct structure of MIL-53(Al)-*np*, we then performed a vibrational analysis on the fully optimized *P*2_1_/*c* structure, and the results of this calculation are shown in Figure 3. It is very clear that the lattice dynamics of the proposed structure are in excellent agreement with those determined experimentally. This also serves to highlight the extreme sensitivity of terahertz dynamics to bulk packing and long-range forces, as the *Cc* and *P*2_1_/*c* structures are very similar—only differing in the orientation of the adsorbed water molecules—but the terahertz spectra are quite distinct. In this case, it is clear that low-frequency vibrational dynamics are a more sensitive probe of structure than powder X-ray methods.

The motions occurring at terahertz frequencies provide insight into the origins of the different spectra and highlight the power of low-frequency vibrational spectroscopy for studying host–guest complexes. It is well-known that terahertz vibrational modes in crystals involve complicated dynamics often including coupling between different molecules, and this is readily apparent in MIL-53(Al)-*np*. In the established *P*2_1_/*c* structure, the terahertz vibrational modes involve a combination of hindered linker rotations (paddle motion) or translations (breathing motion), hindered rotations of the metal nodes, and hindered rotations and translations of the adsorbed water molecules. Within any particular vibrational mode, these dynamics are often coupled to one another, and interestingly, in many cases involve only one pair of symmetry-independent water molecules (although there are cases where both pairs vibrate simultaneously). For example, the lowest-frequency vibrational mode, predicted at 24.9 cm^–1^, involves a coupled rotation of the linker and one pair of adsorbed water molecules (Figure 5). In another instance, the mode predicted at 106.3 cm^–1^ involves an antisymmetric hindered translation of the other pair of water molecules (compared to those that vibrate in the 24.9 cm^–1^ mode) with respect to each other, which is coupled to a bend about the metal node. In both of these illustrative examples, the respective vibrational modes involve motions of only one pair of unique water molecules.

**Figure 5:**
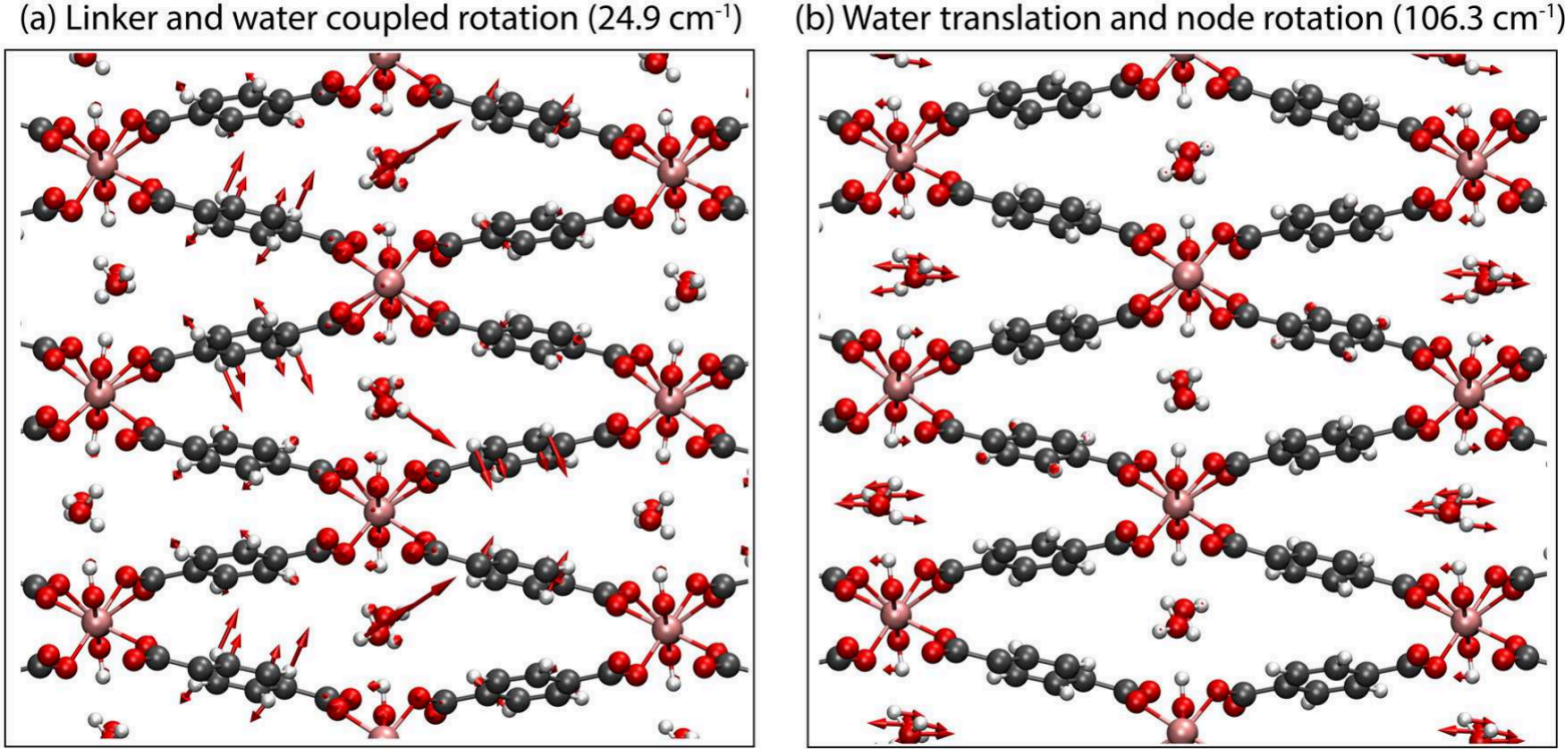
Visualization of the normal mode displacement vectors (red) for two representative modes in MIL-53(Al)-*np*.

In the *Cc* structure, there is only one pair of water molecules in each unit cell. This means that, at the Γ-point, it is not possible to have two different sets of dynamics for two different pairs of water molecules due to translational symmetry. This is one of the leading causes for the differences between the two predicted terahertz spectra. Additionally, the frequencies and associated vibrational mode types are strongly influenced by weak and long-range forces, and thus, different hydrogen bonding patterns will alter the weak forces present within the material, which in turn alters the low-frequency vibrational dynamics.

In conclusion, this study resolves critical uncertainties surrounding the structure of MIL-53(Al)-*np*. While powder X-ray diffraction methods are not able to fully resolve the positions of hydrogen atoms in adsorbed water molecules within the framework, the extreme sensitivity of terahertz vibrational dynamics to long-range forces provides clear contrast that allows us to unambiguously determine the structure of the crystal. In particular, the vibrational modes in the 10–100 cm^–1^ region involve highly coupled dynamics of multiple functional groups, which is the origin of the discussed sensitivity. This work not only contributes to understanding the narrow-pore phase of MIL-53(Al) but demonstrates the broader applicability of terahertz spectroscopy as a powerful tool for elucidating the structures and dynamics of host–guest complexes, with only minor limitations. For example, the wavelength of terahertz radiation implies relatively large beam diameters (ca. 1 mm), and current terahertz assignment methods struggle with disordered guest molecules, but this is an area of active study where such limitations can be overcome. Overall, such insight paves the way for further method development exploring different stimuli, including both temperature and pressure, as well as studying related materials, such as porous solids where diffraction methods fail or produce ambiguous structural results—crucial components to exploiting such materials for advanced applications.

## Supporting Information Available

Optimized structures, trajectories, and animations of normal modes (ZIP)
Experimental methods (PDF)
Transparent Peer Review report available (PDF)
