# Supplementary figures and images for: Terahertz Spectroscopy Unambiguously Determines the Orientation of Guest Water Molecules in a Structurally Elusive Metal–Organic Framework
Source: J Phys Chem Lett. 2024 May 16;15(20):5549–55. doi: 10.1021/acs.jpclett.4c00706 (PMC11129291; doi:10.1021/acs.jpclett.4c00706)

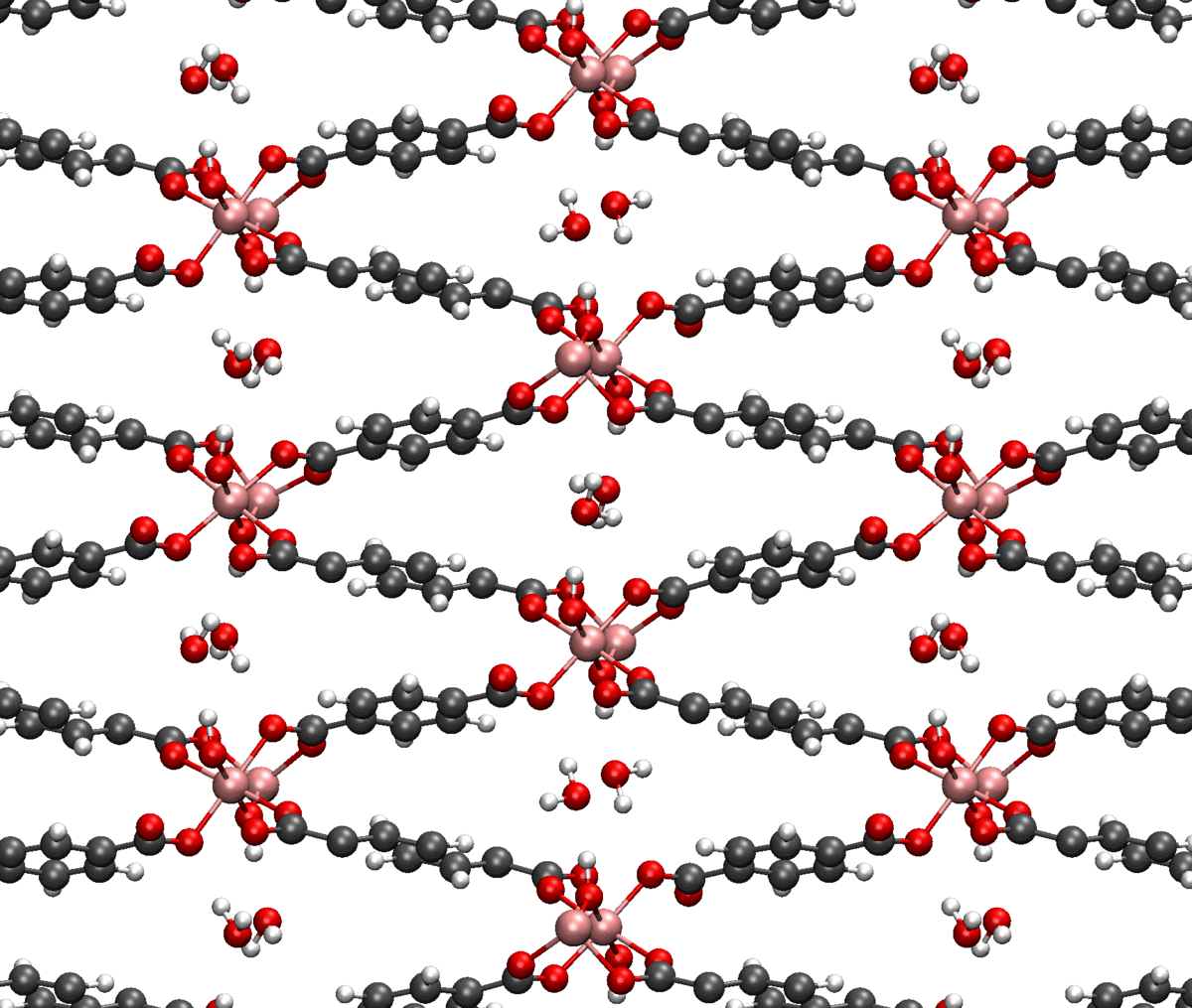

Supplement: Supplementary file 1 — jz4c00706_si_001.zip [file jz4c00706_si_001.zip › 25cm-1.gif]

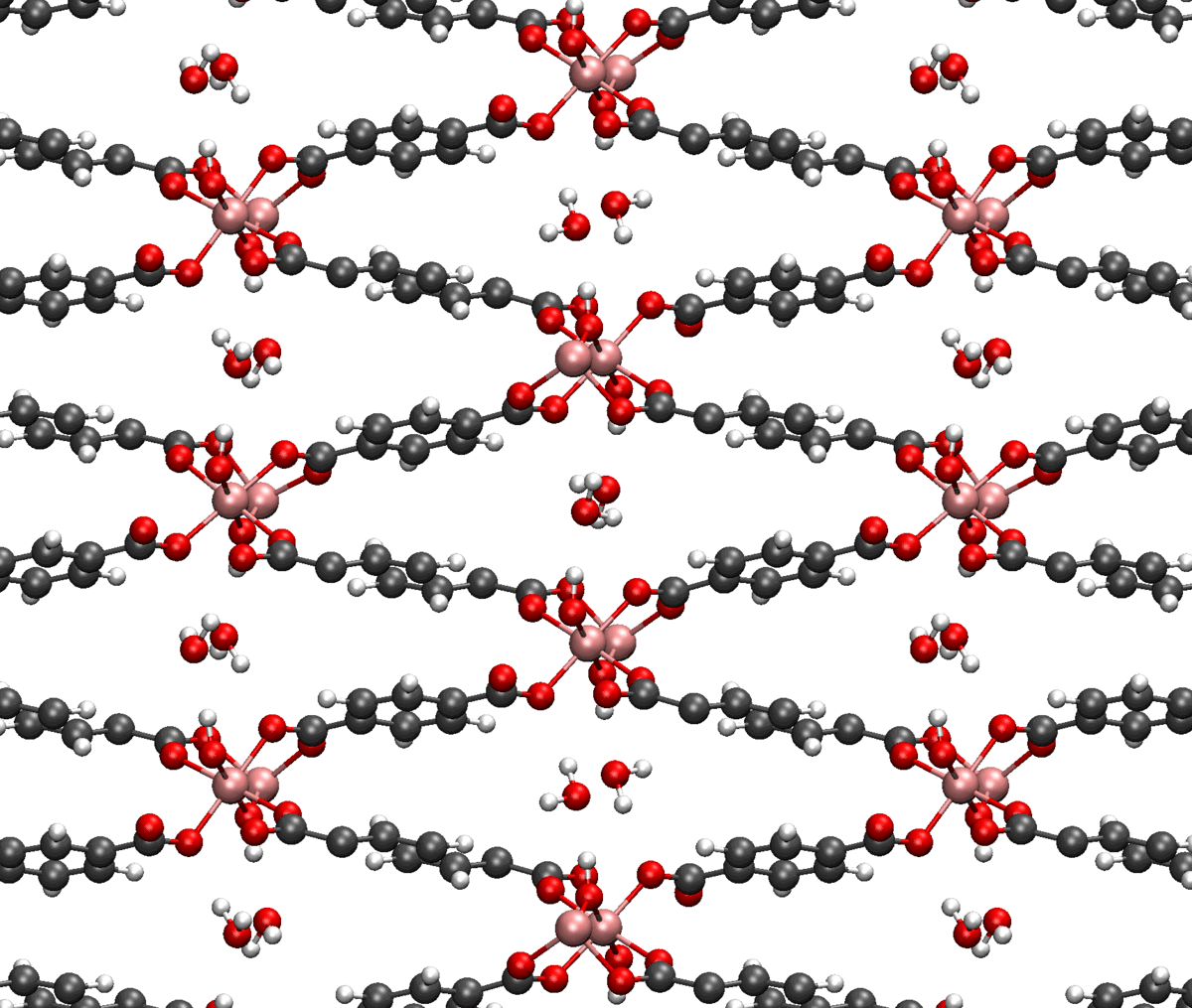

Supplement: Supplementary file 1 — jz4c00706_si_001.zip [file jz4c00706_si_001.zip › 106cm-1.gif]
