# Supplementary material for: Terahertz Spectroscopy Unambiguously Determines the Orientation of Guest Water Molecules in a Structurally Elusive Metal–Organic Framework
Source: J Phys Chem Lett. 2024 May 16;15(20):5549–55. doi: 10.1021/acs.jpclett.4c00706 (PMC11129291; doi:10.1021/acs.jpclett.4c00706)
Supplement: Supplementary file 2 — jz4c00706_si_002.pdf [file jz4c00706_si_002.pdf]

Supporting Information for: Terahertz Spectroscopy Unambiguously Determines the Orientation of Guest Water Molecules in a Structurally Elusive Metal-Organic Framework

Saheed Ajibade,† Luca Catalano,‡,¶ Johanna Kölbel,§ Daniel M. Mittleman,§ and Michael T. Ruggiero*,†,‡

†*Department of Chemistry, University of Vermont, Burlington, Vermont 05405, United States of America*

‡*Department of Chemistry, University of Rochester, Rochester, NY 14627, United States of America*

¶*Department of Life Sciences, University of Modena and Reggio Emilia, 41125 Modena, Italy*

§*School of Engineering, Brown University, Providence, Rhode Island 02912, United States of America*

E-mail: michael.ruggiero@rochester.edu

Experimental Methods

The PXRD measurements were performed using a Rigaku Miniflex powder diffractometer, using Cu K_α radiation ($\lambda = 1.54$ Å). The terahertz time-domain spectroscopy experiments were performed using a commercial Toptica TeraFlash spectrometer. The samples were placed in a cryostat and cooled to 50 K using a closed-cycle liquid helium system. Samples were prepared for the spectroscopy experiments by mixing with polytetrafluoroethylene to a 5% w/w concentration, and then pressing into 13 mm diameter pellets with a thickness of ca. 3 mm. A corresponding blank pellet with the same dimensions was also prepared. For each measurement, a pair of measurements were performed, one for the sample and one for the blank, and involved the collection of 30,000 time-domain waveforms. These waveforms were subsequently Fourier transformed, and the sample and blank frequency-domain spectra were ratioed to produce an absorption spectrum. The reported spectrum is a result of averaging four individual absorption spectra.

Theoretical Methods

CRYSTAL23

The vibrational simulations were performed using the CRYSTAL23 solid-state DFT software package. Prior to the vibrational analysis, the structures were fully-optimized while maintaining the space-group symmetry utilized in the input structure. The simulations were performed with the B3LYP[1] density functional and the 6-311G(2d,2p) basis set.[2,3] The tolerance for the convergence on energy for both the optimization and frequency calculations was set to $\Delta E < 10^{-11} hartree$. Frequencies were calculated within the harmonic approximation using numerical differentiation, using a three-point central difference approach, which corresponds to two displacements per Cartesian axis per atom.[4,5] Infrared intensities were calculated using the Berry Phase method.[6]

AIMD

All AIMD simulations in this work were performed using the solid-state CP2K software package (Version 9.0).[7,8] This was done under the restrictions of the NVT ensemble at 100 K and pressure of 1 atm while taking into account the periodicity of the system.[9] The density functional of choice was the Becke-Lee-Yang-Parr (BLYP) generalized gradient approximation functional.[10,11] The Goedecker-Teter-Hutter (GTH) pseudo-potentials basis sets were utilized, coupled with DFT-D3 dispersion correction as implemented in the CP2K package.[12–15]

References

(1) Becke, A. D. Density-Functional Thermochemistry. Iii. The Role of Exact Exchange. *J. Chem. Phys.* **1993**, *98*, 5648–5652.

(2) Krishnan, R.; Binkley, J. S.; Seeger, R.; Pople, J. A. Self-Consistent Molecular Orbital Methods. Xx. A Basis Set for Correlated Wave Functions. *J. Chem. Phys.* **1980**, *72*, 650–654.

(3) McLean, A. D.; Chandler, G. S. Contracted Gaussian Basis Sets for Molecular Calculations. I. Second Row Atoms, Z=11–18. *J. Chem. Phys.* **1980**, *72*, 5639–5648.

(4) Pascale, F.; Zicovich-Wilson, C. M.; López Gejo, F.; Civalleri, B.; Orlando, R.; Dovesi, R. The Calculation of the Vibrational Frequencies of Crystalline Compounds and Its Implementation in the Crystal Code. *J. Comput. Chem.* **2004**, *25*, 888–897.

(5) Zicovich-Wilson, C. M.; Pascale, F.; Roetti, C.; Saunders, V. R.; Orlando, R.; Dovesi, R. Calculation of the Vibration Frequencies of α-Quartz: The Effect of Hamiltonian and Basis Set. *J. Comput. Chem.* **2004**, *25*, 1873–1881.

(6) Noel, Y.; Zicovich-Wilson, C. M.; Civalleri, B.; D'Arco, Ph.; Dovesi, R. Polarization Properties of ZnO and BeO: An *Ab Initio* Study through the Berry Phase and Wannier Functions Approaches. *Phys. Rev. B* **2001**, *65*, 014111.

(7) Kühne, T. D.; Iannuzzi, M.; Del Ben, M.; Rybkin, V. V.; Seewald, P.; Stein, F.; Laino, T.; Khaliullin, R. Z.; Schütt, O.; Schiffmann, F. et al. CP2K: An electronic structure and molecular dynamics software package - Quickstep: Efficient and accurate electronic structure calculations. *J. Chem. Phys.* **2020**, *152*, 194103.

(8) VandeVondele, J.; Krack, M.; Mohamed, F.; Parrinello, M.; Chassaing, T.; Hutter, J. Quickstep: Fast and Accurate Density Functional Calculations Using a Mixed Gaussian and Plane Waves Approach. *Comput. Phys. Commun.* **2005**, *167*, 103–128.

(9) Nosé, S. A Molecular Dynamics Method for Simulations in the Canonical Ensemble. *Mol. Phys.* **1984**, *52*, 255–268.

(10) Becke, A. D. Density-Functional Exchange-Energy Approximation with Correct Asymptotic Behavior. *Phys. Rev. A* **1988**, *38*, 3098–3100.

(11) Lee, C.; Yang, W.; Parr, R. G. Development of the Colle-Salvetti Correlation-Energy Formula into a Functional of the Electron Density. *Phys. Rev. B* **1988**, *37*, 785–789.

(12) Goedecker, S.; Teter, M.; Hutter, J. Separable Dual-Space Gaussian Pseudopotentials. *Phys. Rev. B* **1996**, *54*, 1703–1710.

(13) Hartwigsen, C.; Goedecker, S.; Hutter, J. Relativistic Separable Dual-Space Gaussian Pseudopotentials from H to Rn. *Phys. Rev. B* **1998**, *58*, 3641–3662.

(14) Grimme, S. Accurate Description of Van Der Waals Complexes by Density Functional Theory Including Empirical Corrections. *J. Comput. Chem.* **2004**, *25*, 1463–1473.

(15) Becke, A. D. Density-Functional Exchange-Energy Approximation with Correct Asymptotic Behavior. *Phys. Rev. A* **1988**, *38*, 3098–3100.
