# Supplementary material for: Terahertz Spectroscopy Unambiguously Determines the Orientation of Guest Water Molecules in a Structurally Elusive Metal–Organic Framework
Source: J Phys Chem Lett. 2024 May 16;15(20):5549–55. doi: 10.1021/acs.jpclett.4c00706 (PMC11129291; doi:10.1021/acs.jpclett.4c00706)
Supplement: Supplementary file 3 — jz4c00706_si_003.pdf [file jz4c00706_si_003.pdf]

jz-2024-00706m.R1

Name: Peer Review Information for "Terahertz Spectroscopy Unambiguously Determines the Orientation of Guest Water Molecules in a Structurally Elusive Metal-Organic Framework"

First Round of Reviewer Comments

Reviewer: 1

Comments to the Author

I thoroughly enjoyed the manuscript showcasing the power of terahertz spectroscopy in the research of porous materials. MOFs, and other porous materials have been extensively studied by diffraction methods, and somewhat by vibrational spectroscopic methods, but a comprehensive application of terahertz is missing. I believe this manuscript with motivate many other physical and materials chemists working in the field to use this method in their research.

Here, terahertz spectroscopy, with its unique sensitivity of local and bulk structure, provided unambiguous solution of the local structure and ordering of water molecules in the inter-pore region of this extremely well studied MOF. These results are indeed novel, and relevant to the MOF community.

I recommend the paper to be accepted as it is.

Reviewer: 2

Comments to the Author

In this paper, the authors have used terahertz (THz) spectroscopy, PXRD and DFT simulations to determine the structure of a well-known flexible MOF, MIL-53 in the presence of water. Although the flexible nature of MIL-53 has been widely studied in other works, the results presented here are very interesting and suggest complementary THz spectroscopy alongside PXRD to characterise host-guest systems for materials with intrinsic structural flexibility. Clearly, the merits of such deliverables are substantial, and I think the paper is publishable after minor corrections.

1. Could the authors expand a bit more on the practical impact of such characterisation flexibility in MOFs in the presence of guests? Speak specifically about wider range of flexible MOFs.

2. Can the authors also expand on the limitations of the tools used? Also, what makes MIL-53 so special? Should we expect similar behaviour in other flexible MOFs? Discuss particularly MOFs for which PXRD methods alone are not able to accurately determine the structures.

3. Can the method be used for other stimuli that affect structural flexibility in MOFs e.g. changes in temperature or mechanical pressure? Please explain in the manuscript.

Reviewer: 3

Comments to the Author

This work demonstrates the application of terahertz spectroscopy for obtaining insight into structural details of complex materials hidden for other experimental techniques. In particular, here a specific structural form of MIL-53 crystals (np-configuration) due to water adsorption has been studied as an interesting example. It is demonstrated that specific vibrational frequency range probed by this technique is very sensitive to weak-long range forces, hence two orientation of water molecules in the MIL-53 cages. This demonstration shows potentials of terahertz spectroscopy for a broader class of material not accessible by other methods. The scopes of this work are well within that covered by JPCL and will certainly be appreciated by the community. The manuscript is very pleasantly written, presents a high quality novel insight. I strongly recommend it for publication as it is. Optionally, the authors may consider making connections to electron-paramagnetic resonance studies of similar systems (see, as just a random example RSC Adv. 2024 Jan 23; 14(6): 4244–4251) providing local insight into location of water in cage structures and highlight differences/similarities.

Author's Response to Peer Review Comments:

Response to the *Journal of Physical Chemistry Letters* Review Comments of: "Terahertz Spectroscopy Unambiguously Determines the Orientation of Guest Water Molecules in a Structurally Elusive Metal-Organic Framework"

Saheed Ajibade, Luca Catalano, Johanna Kölbel, Daniel M. Mittleman, and Michael T. Ruggiero

19th April 2024

We thank all of the reviewers for their time and efforts dedicated to reviewing our manuscript. We have addressed each comment made by the reviewers, and have highlighted changes in the revised manuscript based upon these comments.

Reviewer 1

Recommendation: This paper represents a significant new contribution and should be published as is.

Comments: I thoroughly enjoyed the manuscript showcasing the power of terahertz spectroscopy in the research of porous materials. MOFs, and other porous materials have been extensively studied by diffraction methods, and somewhat by vibrational spectroscopic methods, but a comprehensive application of terahertz is missing. I believe this manuscript with motivate many other physical and materials chemists working in the field to use this method in their research.

Here, terahertz spectroscopy, with its unique sensitivity of local and bulk structure, provided unambiguous solution of the local structure and ordering of water molecules in the inter-pore region of this extremely well studied MOF. These results are indeed novel, and relevant to the MOF community.

I recommend the paper to be accepted as it is.

We thank the Reviewer for their very positive comments.

Reviewer 2

Recommendation: This paper is publishable subject to minor revisions noted. Further review is not needed.

Comments: In this paper, the authors have used terahertz (THz) spectroscopy, PXRD and DFT simulations to determine the structure of a well-known flexible MOF, MIL-53 in the presence of water. Although the flexible nature of MIL-53 has been widely studied in other works, the results presented here are very interesting and suggest complementary THz spectroscopy alongside PXRD to characterise host-guest systems for materials with intrinsic structural flexibility. Clearly, the merits of such deliverables are substantial, and I think the paper is publishable after minor corrections.

We thank the Reviewer for their very positive comments.

1. Could the authors expand a bit more on the practical impact of such characterisation flexibility in MOFs in the presence of guests? Speak specifically about wider range of flexible MOFs.

Certainly, we have added the following sentence to the end of our discussion on the MIL-53 system related to the importance of characterizing framework dynamics in such systems.

> "MIL-53(Al) is an extremely well-studied MOF system that is known to exhibit a range of interesting structural effects. It has been reported to exist in four distinct phases that are strongly influenced by a range of stimuli such as temperature, pressure, and the presence (or absence) of guest molecules. They are: the generic as-synthesized phase; the high-temperature large-pore phase, obtained upon calcination of the as-synthesized form; the low-temperature narrow-pore phase, which results from cooling and adsorption of water molecules at room conditions; and the closed-pore phase (with no guest molecule), which is achieved either by high pressure or low-temperature treatment of the large-pore form. The structural flexibility of MIL-53(Al) has primed it for many varied advanced applications as smart and adaptive porous material. At room temperature, it undergoes a reversible transition from the large-pore to the narrow-pore phase through a breathing effect by adsorption of water molecules into the framework, causing a significant volume fluctuation of up to 40%. This absorption-influenced transition is not limited to water alone, as other guest molecules, including CH_4, N_2, CO, and O_2, have been shown to initiate a similar effect. Additionally, the stability of MIL-53(Al) under high mechanical stress has also been noted, as it has been reported to undergo a pressure-induced phase transition from its large-pore form to the narrow-pore form at high pressures suggesting its suitability as a nano shock absorber. Further, MIL-53(Al) is environmentally and biologically friendly. Overall, the interesting guest-induced framework dynamics in MIL-53(Al) are important to understand from a fundamental standpoint, in order to further design and tune the adsorption behavior of host-guest complexes, and this represents a model system for studying the broader class of flexible MOFs."

2. Can the authors also expand on the limitations of the tools used? Also, what makes MIL-53 so special? Should we expect similar behaviour in other flexible MOFs? Discuss particularly MOFs for which PXRD methods alone are not able to accurately determine the structures.

Certainly, we have made the following modifications to our concluding paragraph,

> "In conclusion, this study resolves critical uncertainties surrounding the structure of MIL-53(Al)-*np*. While powder X-ray diffraction methods are not able to fully resolve the positions of hydrogen atoms in adsorbed water molecules within the framework, the extreme sensitivity of terahertz vibrational dynamics to long-range forces provides clear contrast that allows us to unambiguously determine the structure of the crystal. In particular, the vibrational modes in the 10–100 cm^{-1} region involve highly-coupled dynamics of multiple functional groups, which is the origin of the discussed sensitivity. This work not only contributes to understanding the narrow-pore phase of MIL-53(Al), but demonstrates the broader applicability of terahertz spectroscopy as a powerful tool for elucidating the structures and dynamics of host-guest complexes, with only minor limitations. For example, the wavelength of terahertz radiation implies relatively large beam diameters (ca. 1mm), and current terahertz assignment methods struggle with disordered guest molecules, but this is an area of active study where such limitations can be overcome. Overall, such insight paves the way for further method development exploring different stimuli, including both temperature and pressure, as well as studying related materials, such as porous solids where diffraction methods fail or produce ambiguous structural results – crucial components to exploiting such materials for advanced applications."

3. Can the method be used for other stimuli that affect structural flexibility in MOFs e.g. changes in temperature or mechanical pressure? Please explain in the manuscript.

We agree that explicitly stating that terahertz spectroscopy can be performed with other external stimuli is important, and have made the following two changes to our manuscript,

> "The long-range nature of the forces involved in terahertz dynamics makes the technique a powerful tool for studying structural changes in crystalline solids. This is because any alteration of the bulk packing of molecules in a crystal will re-configure the set of weak forces that act on individual molecules, which in turn results in a restructuring of the low-frequency vibrational dynamics. This sensitivity has been demonstrated extensively for the detection of different polymorphic materials, for quantifying crystalline content in amorphous materials, and, recently, as a powerful complement in crystal structure prediction studies. Porous materials have also been studied with terahertz spectroscopy, including in situ studies of gas adsorption in organic clathrates as a function of both temperature and (gaseous) pressure, which can offer quantitative measurements of loading fraction due to the dramatic changes in the spectra as gas adsorbs into the material."

and in our concluding remarks,

> "In conclusion, this study resolves critical uncertainties surrounding the structure of MIL-53(Al)-*np*. While powder X-ray diffraction methods are not able to fully resolve the positions of hydrogen atoms in adsorbed water molecules within the framework, the extreme sensitivity of terahertz vibrational dynamics to long-range forces provides clear contrast that allows us to unambiguously determine the structure of the crystal. In particular, the vibrational modes in the 10–100 cm^{-1} region involve highly-coupled dynamics of multiple functional groups, which is the origin of the discussed sensitivity. This work not only contributes to understanding the narrow-pore phase of MIL-53(Al), but demonstrates the broader applicability of terahertz spectroscopy as a powerful tool for elucidating the structures and dynamics of host-guest complexes, with only minor limitations. For example, the wavelength of terahertz radiation implies relatively large beam diameters (ca. 1mm), and current terahertz assignment methods struggle with disordered guest molecules, but this is an area of active study where such limitations can be overcome. Overall, such insight paves the way for further method development exploring different stimuli, including both temperature and pressure, as well as studying related materials, such as porous solids where diffraction methods fail or produce ambiguous structural results – crucial components to exploiting such materials for advanced applications."

Reviewer 3

Recommendation: This paper represents a significant new contribution and should be published as is.

Comments: This work demonstrates the application of terahertz spectroscopy for obtaining insight into structural details of complex materials hidden for other experimental techniques. In particular, here a specific structural form of MIL-53 crystals (np-configuration) due to water adsorption has been studied as an interesting example. It is demonstrated that specific vibrational frequency range probed by this technique is very sensitive to weak-long range forces, hence two orientation of water molecules in the MIL-53 cages. This demonstration shows potentials of terahertz spectroscopy for a broader class of material not accessible by other methods. The scopes of this work are well within that covered by JPCL and will certainly be appreciated by the community. The manuscript is very pleasantly written, presents a high quality novel insight. I strongly recommend it for publication as it is. Optionally, the authors may consider making connections to electron-paramagnetic resonance studies of similar systems (see, as just a random example RSC Adv. 2024 Jan 23; 14(6): 4244-4251) providing local insight into location of water in cage structures and highlight differences/similarities.

We thank the Reviewer for their very positive comments.
